# An Act

**Published:** 1901-03

**Authors:** 


### LEGISLATION.

### AN ACT

Entitled "An Act providing for a Meat and Milk Inspector in cities having five thousand inhabitants or over within the State of Montana, and prescribing the duties and powers of such inspector, and to regulate the sale of meat and milk and live domestic animals intended for food purposes and prescribing penalties for the violation of the provisions of this Act."

*Be it enacted by the Legislative Assembly of the State of Montana:*

Section 1. The office of Meat and Milk Inspector is hereby created for cities having a population 5000 inhabitants or over within the State of Montana, and immediately after the taking effect of this Act such cities shall appoint a Meat and Milk Inspector, whose compensation shall be borne by the said cities, and shall be such as will secure the services of some competent and qualified person, who shall take an oath of office to faithfully perform the duties of his office and to execute an official bond to the said city in the sum of $5000. No person shall be appointed to the office of Meat and Milk Inspector unless he is a graduate in good standing of some regular and reputable veterinary medical college recognized by the American Veterinary Medical Association and admitted to practice within the State of Montana, and before such appointment he shall be required to exhibit his diploma as such graduate.

Section 2. It shall be the duty of the city council of all cities having a population required by this Act to designate some place or places in or adjacent to the city where the cattle, sheep, or swine or other domestic animals intended for slaughter, sale, and consumption for food in said city shall be brought for inspection on the hoof or where the meat of any such animal or animals shall be brought for inspection, which inspection shall be made without any unreasonable delay, and no fee or charge shall be made against or demanded of the owner or person who shall present any such animal or animals or meat intended for food for such inspection, but the same shall be inspected free of any expense whatever to the owner of said food animals intended for meat for or on account of the services of such inspector. And it is hereby made the duty of such inspector to keep a correct record in a suitable and substantial book provided by the municipality for that purpose, in which he shall record the name, place of residence, and post-office address of the owner or owners of all such animals intended for food and the carcasses or parts of carcasses presented for inspection, together with brands and marks and a full description thereof.

Subdivision 2. The rules, regulations, and methods of inspection adopted by the Bureau of Animal Industry, conducted by the United States Government, shall be taken as the standard of meat-inspection and shall be followed as closely as may be consistent by the Meat and Milk Inspectors appointed by the said cities.

Section 3. Subdivision 1. All animals intended to be slaughtered for meat for human consumption shall be examined both before and after slaughter.

Subdivision 2. The carcasses of all animals so inspected on hoof shall be properly tagged and marked with the official tag or mark of such municipality before being offered for sale, and such carcass or parts of carcass of any of the animals mentioned in this Act, where the animals shall not have been presented for inspection on hoof before being slaughtered, shall be inspected before being offered or exposed for sale, and such carcass or carcasses or meat as shall be found upon such inspection and examination to be wholesome and fit for food shall be marked as above mentioned by the inspector with a tag similar in form and character to that used by the Bureau of Animal Industry, Department of Agriculture, which tag shall be adopted and designated by the city council of such municipality as the city stamp or certificate for the designation of wholesome and healthy meat: Provided, that nothing herein contained shall be so construed as to prevent any person from slaughtering any healthy animal the meat of which is intended for his own use or that of his family, but shall not be offered for sale for public consumption: Provided further, however, that nothing in this Act shall be so construed as to permit any person to slaughter and offer for sale any meat or meats intended for domestic consumption before being inspected on the hoof, excepting where such slaughter may be conducted in a locality inaccessible to said municipal meat and milk inspector.

Section 4. It shall be the duty of the Inspector to make inspection of the meats, carcasses, and animals mentioned in this Act which may be presented for inspection at the place or places designated by the municipal council and keep the record aforesaid in the manner herein provided, which inspection or inspections shall be made by him as soon as possible and without unreasonable or unnecessary delay, and he shall attach to all such meat so inspected and examined and found suitable and wholesome and fit for consumption a tag such as is prescribed above, indicating that fact.

Section 5. The Meat and Milk Inspectors appointed by said cities shall have the right to condemn any meat, carcass or carcasses, or parts thereof, of all cattle, sheep, swine, or other domestic animal intended for food which they find after examination to be unfit for food, and it shall be said inspector's duty to destroy all such condemned meat by slashing said meat and muscular tissue deeply in numerous places with a knife, into which incisions he shall then pour sufficient kerosene to taint such meat and make it impossible to be used for human consumption.

Section 6. It shall be unlawful to sell or offer for sale, buy or offer to buy, take or give away for the purpose of food, any animal suffering from hog cholera, swine plague, charbon or anthrax, rabies, malignant epizoötic catarrh, pyæmia and septicæmia, mange or scab in advanced stages; advanced stages of actinomycosis or lumpy jaw, inflammation of the lungs, the intestines, or the peritoneum; Texas fever; extensive or generalized tuberculosis; animals in an advanced stage of pregnancy or which have recently given birth to young; any disease or injury causing elevation of temperature or affecting the system of the animal to a degree which would make the flesh unfit for human food; any organ or part of the carcass which is badly bruised or affected by tuberculosis, actinomycosis, cancer, abscess, suppurating sore or tapeworm cysts; animals too young and immature to produce wholesome food; animals too emaciated and anæmic to produce wholesome meat; distemper, glanders, and farcy or any other malignant disorder; acute inflammatory lameness and extensive fistula.

Section 7. Any person or persons, company or corporation who shall sell or offer for sale, buy or offer to buy, take or give away within the limits of said city any carcass or carcasses or portion thereof of any cattle, sheep, or swine or other domestic animal which has not been inspected and tagged as herein required except as herein stated, or shall violate any of the provisions of this Act, shall be guilty of a misdemeanor and upon conviction thereof be punished by a fine of not less than fifty dollars nor more than five hundred dollars for each separate offense.

Section 8. Nothing in this Act or any paragraph thereof shall be so construed as to interfere with the offering for sale of any meats bearing a stamp or tag indicating that the same has been inspected by the United States Bureau of Animal Industry or of any State, county, or municipal meat inspector where regulations equal to those prescribed herein are observed.

Section 9. It shall be the duty of the said Meat and Milk Inspector to inspect each dairy supplying milk to such municipality not less than once in every month of the calendar year ; and it shall be the duty of such inspector to issue to each person or persons or corporation supplying milk to the citizens of said municipality a certificate of health every ninety days, which certificate of health shall include a certificate of the sanitary conditions of said dairy, and must specify each and every cow within said dairy from which milk is supplied to the public.

Section 10. It shall be a misdemeanor for any dairyman, person, persons, or corporation to feed unwholesome food of whatsoever character, and for each offense the owner or owners of such dairy shall be fined not less than fifty dollars nor more than five hundred dollars. Each dairyman, person, persons, or corporation supplying milk to the public must have for each cow a certificate of health, including the tuberculin test made by said inspector stating that said cow is free from tuberculosis or consumption or any infectious or other disease whatsoever.

Subdivision 2. Any dairyman having in his own family or among his employés, or about his premises, anyone suffering from diphtheria, scarlet fever, typhoid fever, or any infectious or contagious disease that may or might contaminate said milk, is prohibited from selling said milk to the public for such period as such disease or diseases exist in his or her family or among his or her employés and for twenty days after said disease has subsided and said inspector has satisfied himself that such premises have been thoroughly disinfected and has issued a certificate so stating.

Section 11. The milk supplied by said dairies or purveyors of milk shall not contain less than 3 per centum of butter fats and shall come up to a normal and accepted specific gravity test for milk.

Section 12. When, in the belief of the said Meat and Milk

Inspector, proper cleanliness of the buckets, pans, cans, and other utensils used about, accumulating, handling, or marketing of said milk is not up to the proper standard, it shall be the inspector's duty to destroy such milk and prohibit said dealer, person or persons, or corporations from selling milk until such time as proper methods of cleanliness and precaution are used in its handling.

Section 13. All persons or corporations engaged in the dairy business and supplying milk to the citizens of said municipalities shall keep their barns and stables free from filth or dirt or rubbish or manure likely to harbor or favor the growth of disease-producing germs within or about their stables likely to be carried within said milk.

Section 14. It shall be the duty of said inspector to stop at any time he may deem fit any dairy wagon, cart, or vehicle of any character, person or persons hauling, carrying, or conveying milk that is intended for public consumption, and there and then take cognizance of any irregularity in such milk or the method of handling or distributing said milk. He shall ascertain if it is not up to the regular standard, or the recognized standard, to which milk should come, and if he finds said milk deficient in any of its nutritive qualities or to contain any drug or preservative or coloring-matter or other extraneous matter he shall there and then condemn such milk; and such dairyman or milkman, or person or persons, or corporation whose product shall be condemned shall be prohibited from selling any milk until they shall have received a written permit from said inspector permitting him so to do: Provided, that such inspector shall, if requested by such dairyman, take from the same can of milk from which he shall have taken any quantity of milk for the purpose of testing the same at least one pint of such milk, place the same in a bottle, adding sufficient formaldehyde to such milk to prevent fermentation, and seal and mark such milk in such manner as to identify the same and deliver the same to such dairyman, who may have said milk analyzed and tested by any chemist competent to test and analyze such milk, in order that said dairyman may ascertain the correctness of the inspector's analysis of such milk: Provided, that at the time of taking such specimen for said dairyman and for said inspector a third specimen shall be taken by said inspector, consisting of not less than one pint of said milk, which shall be taken from the same can from which the other specimens were taken, which must be sealed in the presence of said dairyman, person, persons, or agent, and which said specimen shall be immediately forwarded to the chemist of the Agricultural Experiment Station at Bozeman for analysis, and said chemist of said Agricultural Experiment Station shall in all cases, where so requested by said dairyman, person or persons, or corporation, act as umpire in said chemical analysis.

Section 15. Any resident of the State of Montana to whose knowledge or observation comes the fact that any dairyman, person or persons, or corporation is supplying milk from any diseased cattle, or cattle fed on stable bedding, stable refuse, or other improper food of any character whatsoever, it shall be his duty to at once notify said inspector of such municipality, who shall at once visit the premises or place indicated, and if he finds the complaint to be true it shall then be the said inspector's duty to at once prohibit the further selling of the product of such dairy or dealer and at once file the information against said dairyman, person, persons, corporation, or dealer in the nearest court.

Section 16. This Act shall apply to all products of the dairy in any municipality to which this Act applies where sold in the State, county, or any municipality to which the district covered by said inspector belongs.

Section 17. It shall be a misdemeanor to adulterate milk in any manner whatsoever in a way likely to produce an unwholesome change in said milk, or disease to the consumer, and such milk shall be prohibited from exposure to sale, and any violation of this section shall be a misdemeanor and be punished as is herein mentioned within the meaning of this Act. The use of any product or any unnatural method whatsoever for the preservation or changing of milk excepting by pasteurizing or sterilization shall be a misdemeanor and be punished as is provided for in this Act.

Section 18. Any city in the State of Montana having a population of less than five thousand inhabitants shall have the option of adopting the sanitary provisions of this Act.

Section 19. Any violation of the provisions of this Act shall be a misdemeanor and shall be punished by a fine of not less than fifty dollars or more than five hundred dollars for each separate offense.

Section 20. This Act shall take effect and be in force from and after the first day of May, 1901.

Section 21. All Acts and parts of Acts in conflict herewith are hereby repealed.

